# Value Conflict, Lack of Rewards, and Sense of Community as Psychosocial Risk Factors of Burnout in Communication Professionals (Press, Radio, and Television)

**DOI:** 10.3390/ijerph18020365

**Published:** 2021-01-06

**Authors:** Santiago Gascón, Ricardo Fueyo-Díaz, Luis Borao, Michael P. Leiter, Álvaro Fanlo-Zarazaga, Bárbara Oliván-Blázquez, Alejandra Aguilar-Latorre

**Affiliations:** 1 Department of Psychology and Sociology, University of Zaragoza, 50009 Zaragoza, Spain; sgascon@unizar.es (S.G.); rfueyo@unizar.es (R.F.-D.); lborao@unizar.es (L.B.); 2 Department of Psychology, Acadia University, Wolfville, NS B4P 2R6, Canada; michael.leiter@acadiau.ca; 3 Health Science Institute Aragón (IACS), 50009 Zaragoza, Spain; alvarofz@hotmail.com; 4 Institute for Health Research Aragón (IIS Aragón), 50009 Zaragoza, Spain; aaguilar@iisaragon.es

**Keywords:** communication professionals, burnout, values, psychosocial risks

## Abstract

Journalists are at particular risk of work-related stress and burnout. The objective of this study is to describe and analyze the principal factors involved in the appearance of burnout in communication professionals, as well as the possible interactions between them and with self-reported health, and to observe whether the variables involved are the same in different types of environments. To achieve this objective, 292 participants answered the following measurement instruments: Demographic and labor datasheet; Maslach Burnout Inventory (MBI General survey); Areas of Worklife Scale (AWS); and General Health Questionnaire (GHQ -12). The results were the following: Emotional Exhaustion (EE) shows direct correlation and statistical significance with the other two burnout dimensions, Depersonalization (DP) and Personal Accomplishment (PA), also with health perception variables and inverse and statistical significance with the workload, control, rewards, community, fairness, and values. A multiple linear regression model shows workload and values as inverse EE predictors, which confirms a burnout process in which EE contributes as the main dimension in DP and is shown to be a precursor of PA, itself. When comparing different types of media, journalists who work in institutional press offices presented significantly lower scores in PA and higher in control, rewards, community, justice, and values. Therefore, further research should be carried out in order to analyze the protective role of these variables regarding PA and burnout.

## 1. Introduction

Information professionals have been identified as a high-risk sector for health problems derived from work stress and burnout [1]. To a great extent, some distinctive aspects have been considered with respect to other professions as the main cause of this: the profession has suffered dizzying changes in the last decades. Consequently, these have produced drastic cuts in the workforce, as well as an overload in the quantity and variety of tasks which require constant updating with new technologies by the remaining personnel [1,2].

### 1.1. Stress in Journalists

Initial research in this field was focused on the posttraumatic stress symptomatology suffered by war correspondents, or catastrophe witnesses/informants [3]; however, these dramatic situations are no longer representative of the typical activity of such professionals.

Psychosocial factors should be understood as those that are present in a certain work environment, interacting between the person and the working conditions, and that correspond to the content and the compliance of the task and the organization itself [4]. These factors may become risks when they trigger maladjustment or psychophysiological stress responses [5,6]. The list of factors that may become risks is quite extensive and various aspects have been highlighted: organizational (hierarchy, resource management, supervision, leadership, working environment) [7]; employment conditions (stability, rotation, use of skills); task (mental and physical load, control possibility) [8]; relations (with managers, colleagues or clients).

Information professionals, due to many causes (continuous changes in formats, technology, demands, instability at work, or living their career in a vocational way), can take on a wide variety of responsibilities. All the while, the times are marked by the current climate of accepting long and changeable schedules with little stability [4]. Overload doesn’t seem to be the only variable that explains the wear in this and other professions [9], the psychosocial factors can vary according to the type of media (newspaper, radio, television, news agency, etc.) and depending on the format (direct, daily press, etc.). In an environment where changing reality and immediacy predominate, it was to be expected that the variable “possibility of control over the task” had a bigger predicted power of stress levels, especially, in audiovisual environments [2,6,8].

To a greater or lesser degree, in these kinds of environments it is usual to work in direct contact with people: interviewees, collaborators, listeners that interact and demand. On the other hand, the need for teamwork between reporters, editors, documentary makers, producers, managers, etc., is common, creating a relationship of dependency in an activity where every member is essential and on which each should work to the best of their abilities within a short timeframe. The interpersonal relations between workers, partners, and managers, or between professionals in the outside environment, are the most important and could become both a protective factor but a cause of stress too and gain special importance in critical situations of harassment when psychological aggressions are used as a method to force people into abandoning the field [10,11].

A central aspect in different professions is recognition, feedback that is necessary, but difficult to obtain in any work environment, and even more so in an environment ruled by immediacy. In addition to recognition and the sense perceived of justice, a work-life area that gains importance is the relative congruence or incongruence between personal principles and those of the organization [10,12]. It is common that an employee starts their career with a strong commitment to their personal aims and those of the company and, for different reasons, distances themselves over time [9]. We must add to all these factors that journalists need to be up to date with news and technology upgrades which quickly reach obsoletion [5,6]. Beyond this, they also must show or spread content, independently of their opinion, sometimes far from their beliefs or ideologies, and that could arouse criticism or rejection in an audience that is in constant interaction [10].

### 1.2. Chronic Stress or Burnout

The concept of burnout has generated controversy for decades, including when the WHO typified it as a work-related pathology [4]. Some authors, such as Pines and Aronson, have considered it a one-dimensional construct, characterized by exhaustion [13], and other researchers [14] have considered it a complex syndrome composed of the dimensions of Emotional Exhaustion (EE), Depersonalization –or Cynicism (DP)–, and a diminished sense of Personal Accomplishment (PA). In any case, this chronic condition has dramatic consequences for employees’ health: depression, anxiety, immunological, cardiovascular, digestive problems, etc., as well as adverse effects for the companies: absenteeism, job abandonment, errors, etc. [15].

Since Maslach and Jackson (1981) generated the “Maslach Burnout Inventory” (MBI) [14] to measure the three subscales of EE, DP, and PA, countless studies have been carried out in different professions and multiple countries [10,16]. Later, Leiter and Maslach offered a model based on the variables or “Areas of Work Life” (AWL) [17], which could contribute to burnout experience. The proposed areas are workload, control, rewards, community, fairness, and values, and are based on the results of several surveys on labor-related stress.

“Workload” has shown, in multiple investigations, its direct relationship with EE [18]. Closely related to this, the fact that the worker has “control” over the task can reduce EE [9,10]. Other variables can be protective or deleterious irrespective of high overload situations. In this way, “rewards”—recognition, or intrinsic satisfaction derived from work–, are a moderating variable in the process of burnout at work [19]. “Community” refers to both interpersonal support relationships, in a positive sense, and conflicts with managers or colleagues, on the negative side [11,17]. The “fairness” area describes the perception of equity felt by employees within their organization [17]. Finally, the area of “values” reflects the compatibility or conflict between the values of the individual and those of the organization, as well as the great motivational potential that the aims and hopes of employees have when they coincide with the goals of their company [20].

Surveys from the “Areas of Worklife (AWL)” model show that it explains a significant percentage of the burnout variance in differing productive sectors around the world [9,10,21], and is considered an optimal instrument to evaluate psychosocial risk factors before designing future interventions.

Although some intervention methods aimed at the individual have obtained empirical support in managing work stress, they are not fully effective on their own and their benefits tend to decline over time [22]. Therefore, it is usually preferable to implement intervention methods at the organizational level based on the strengths found in an initial evaluation, focusing on the elimination or modification of as many causes as possible to reduce stress and prevent its reappearance in the future [23].

The objective of this research was to describe and analyze the main factors involved in the appearance of burnout in communication professionals in relation to areas of work-life, sociodemographic, occupational, and health variables, as well as observing possible differences in these variables according to different types of media, in order to design intervention programs in the media to establish a permanent preventive culture. The hypothesis was that the workload would be, as in many other professions, the area that would mainly predict the EE rates. Similarly, it was expected that the variable “control”, or lack of control over the task, would help explain the three dimensions of burnout since it was assumed that journalists must deal with multiple and different tasks and that the latest news is not something that can be controlled by them.

## 2. Materials and Methods

A transversal descriptive study developed throughout the months of September to December of 2019. This first analysis of psychosocial factors in the media is part of a wider study. The data were used to design and implement a model of permanent prevention based on the clarification of the company’s values, the implementation of a transparent justice system, and the distribution of rewards. The evaluation of possible improvements and their maintenance, through the model application in a newspaper, a radio station, and a television studio, has not yet been analyzed.

### 2.1. Participants

Approximately 1200 communication professionals work in Aragon. A sample size was reached, estimated according to the evaluation criterion of the 10:2 recommended ratio for the number of subjects and the number of test items included in the confirmatory factor analysis. Therefore, psychometric adequacy was provided for the analysis [24]. A total of 830 people were invited to participate in conduct their work in the communication media in this Spanish region. Information sessions were held in the workplaces, contacting those interested in participating and distributing information brochures. E-mails were sent from the Human Resources offices. Additionally, contact was made with the Aragon Journalists Association to spread the information through their usual channels amongst their members.

The inclusion criteria were (a) subjects over 18; (b) they understand spoken and written Spanish; (c) they give their informed consent; (d) and they are working actively in any media in the autonomous community of Aragon (Spain). Exclusion criteria were the failure to give consent and incomplete questionnaires.

A total of 323 professionals showed their availability to participate in the survey. Finally, after applying the inclusion criteria, 292 professionals were considered. Three working groups (with a total of 13 professionals) were created to discuss and interpret the final results, in light of their experience, and to make proposals on the preventive design to be implemented in their working environments.

### 2.2. Instruments

Demographic and labor datasheet. The following variables were collected: sociodemographic variables (gender, age, and cohabitation) and work variables (working time in the field of information, work contract type, media type (newspaper, radio, television, others) and profession (manager, drafting/screenwriter, reporter, institutional communication, camera photography, layout artist, publicist, administrator, and others).

Maslach Burnout Inventory (MBI General survey, Maslach, Jackson and Leiter, 1996) [14]. It provides information about the three dimensions that constitute their theoretical model of burnout: Emotional Exhaustion (EE), Depersonalization (DP), and Personal Accomplishment (PA). The questionnaire was validated in Spanish by Gil Monte (2002) [25], who found the reliability of α: 0.89 for the EE, 0.67 for DP, and 0.74 for PA. The 16 items are rated using a Likert scale on which the frequency with which the described situation has been experienced, from 0 to 6, is indicated. Thus, for example, to the question “I feel emotionally exhausted at work”, the answer should inform of the frequency with which it happens, ranging from 0, never, to 6, daily. Low scores on professional efficiency and high scores in exhaustion and cynicism mean perceptions of being “more burned by work”.

Areas of Worklife Scale (AWS, Leiter and Maslach, 1999) [17]. It consists of 29 items that evaluate the degree of fit or mismatch that the professional perceives about the main variables of their work area environment. Those areas are manageable workload, the possibility of control over the tasks, existence or non-existence of intrinsic rewards at work, sense of community, perceived justice or fairness, and values (concordance between own values and those of the organization). The questionnaire requests an answer to the items with which the subject agrees, to some extent, in each of the statements. For example: in the statement “I don’t have time to do the work that must be done”, the subject could answer with 1 (strongly disagree) to 5 (strongly agree). The version validated for Spanish language was used [26], which gained a Cronbach’s Alpha ranging from 0.75 to 0.88: Workload = 0.80; Control = 0.87; Reward = 0.84; Community = 0.75; Fairness = 0.88; and Values = 0.85.

General Health Questionnaire (GHQ -12, Goldberg and Williams, 1988) [27]. It values general aspects of cognitive function and psychological symptoms and is used in psychiatric populations to obtain the general assessment of cognitive functioning in recent weeks. In their short version, the 12 items report four subscales: somatic symptoms, anxiety and insomnia, social dysfunction, and depression. Using the Likert scale between 0 and 3, 0 indicating always and 3 never. The validated Spanish version [28] revealed a Cronbach’s alpha of 0.76 on the global scale.

### 2.3. Statistical Analysis

The sample presented a non-normal distribution on the quantitative variables; given that the Kolmogorov-Smirnov statistic was used, the p-values of all these variables were lower than 0.05. For this reason, non-parametric statistics were chosen for the correlation analysis—Spearman’s Rho—and comparison of means between groups—U Mann-Whitney and Kruskal-Wallis. The correlation between the three burnout dimensions (EE, DP, PA) and other variables: age, experience, health perception, and work environment were analyzed. The comparisons were grouped by gender, by type of contract (permeant, temporary, self-employed), and by the environment in which the participant was working.

A first multivariate model was developed for each of the three burnout dimensions. The independent variables were added into the regression model with a stepwise method [29] with which a final model was obtained for each facet of burnout. Regarding the regression on EE, the workload was added in Step 1 and the other areas in Step 2. This is because the workload is always closely correlated with EE and must always be included [30]. As for the regression on DP, EE was added at Step 1, and subsequently the areas of work-life; and for the regression on PA, DP was inserted at Step 1 and after that, the areas of work-life. This is because the three aspects of burnout define a syndrome. They are related to one another and those relationships among the three parts of burnout need to be included in the analysis. Subsequently, a multivariate scales model was developed to identify the strength of the associations among the variables. The independent variables were put into the regression model with a stepwise method. Linear regression was carried out since the following conditions were met: the residuals of the model had a normal distribution (above all, because the sample size is very high and, with the central limit theorem, any distribution with constant mean and variance—if it has a large enough sample size—has a normally distributed mean); and had a finite mean and constant variance.

Interactions in a multiple regression analysis were also explored, in order to investigate whether workload has a different effect on the outcome (EE, DP, and PA) depending on the values of other independent variables (Areas of Worklife, AWS).

### 2.4. Ethical Questions

The research was conducted in accordance with the Helsinki Declaration of 1975, as revised in 2008. Every participant subject previously provided informed consent for access to the questionnaire and the data was anonymized. The project received the approval of the Aragon Occupational Safety and Health Institute (ISSLA, Government of Aragon) (Code 2018/0507).

## 3. Results

### 3.1. Description of Sample

The sample consisted of 292 subjects: 145 males (49.7%) and 147 females (50.3%). The mean age was 43.45 years (SD: 9.56), age range (22–67). Of them, 76.6% were living with a stable partner. In terms of the work variables, two-thirds of the sample had more than 12 years’ experience in the sector, more than half had a permanent contract, and presented mean low levels of exhaustion, cynicism, and lack of personal accomplishment (Table 1).

### 3.2. Burnout’s Dimensions, Areas of Work-Life, and Health

In terms of the correlations obtained between the burnout dimensions and the main variables of investigation (Table 2), EE shows direct correlation and statistical significance with the other two burnout dimensions (DP and PA), as well as with health perception variables and an inverse correlation and statistical significance with the workload, control, rewards, community, fairness, and values. DP shows direct significant correlations with the health perception variable, with the other burnout dimensions (EE and PA), and an inverse significant correlation with control, health perception, rewards, community, fairness, and values.

A sequential model of burnout was analyzed, using multiple linear regression, verifying that the workload variable was the main predictor of EE (−1.297), together with values (−1.072). EE turned out to be the variable that contributed the most to DP (0.132), while the dimensions of DP and EE were statistically significant predictors of PA (0.355 and 0.287, respectively) [17].

The results of the linear regression model, taking into account each dimension of burnout separately as dependent variables showed that, with respect to the dimension of EE, workload, rewards, values, and community were shown as inverse coefficients predictors, and that for each point of the workload variable it decreases by almost a point (−0.941) EE. Furthermore, for each point of rewards, values, and community, EE decreased by approximately half a point (0.648, 0.538, and 0.491, respectively). The model accounts for 57.1% of the global variance (Table 3). Taking DP as a dependent variable, reward and community are inverse coefficients of the depersonalization predictors, so that with each point the community and reward variables, depersonalization decreased 0.30 and 0.19 points. The model accounts for 23.3% of the variance. Finally, when taking PA as a dependent variable, the following variables were shown to be inverse predicting coefficients: congruence values, rewards, community, and manageable load. With each point on the variables—values, rewards, community, and load, 0.398, 0.334, 0.260, and 0.155 points, respectively—, a decrease in lack of personal accomplishment was found. The model accounts for 46.3% of the variance.

**Figure 1:**
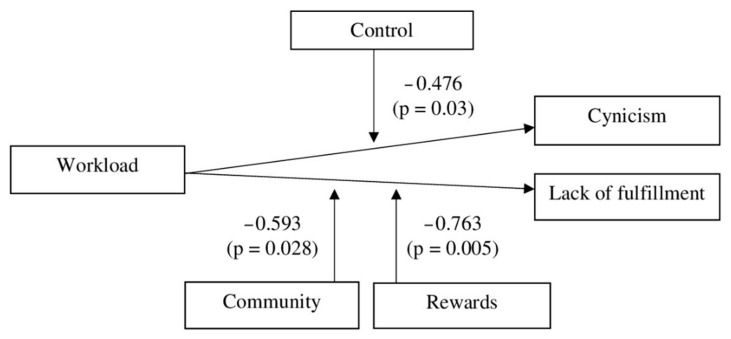
Significant interactions.

Regarding the interactions in a multiple regression analysis, the workload is related to DP in a different way when employees have a high or low score in control (−0.476, *p* = 0.030). Additionally, the workload is related to PA in a different way when the employees have a high or low score in rewards (−0.763, *p* = 0.005). Finally, the workload is associated with PA in a different way when the employees have a high or low score in the community (−0.593, *p* = 0.028). We created Figure 1 to show those significant interactions. The rest of the interactions were not meaningful.

In terms of the different types of media (newspapers, radio, TV, institutional press) in the study (as is shown in Table 4), significant differences were found relating to the following variables: PA, control, rewards, community, fairness, and values. The institutional press workers showed significantly higher scores in control, rewards, community, justice, and values, and lower scores in PA compared to newspapers, radio, or TV workers, although there were no significant differences comparing workload among the different types of media.

## 4. Discussion

As evidenced by several investigations conducted in different work environments, our study with journalists showed that the higher the manageable load index, the lower the level of EE [9,31]. However, the possibility of exercising “control” over the task itself was not shown to be an explanatory variable for any of the burnout dimensions, as we hypothesized before this study began. This hypothesis was plausible, since communication professionals work with an uncontrollable subject—the latest news—and, frequently, they are forced to change their schedules and calendars because of it. Other areas contributed to depletion levels, namely, as the “congruence of values” increases, exhaustion levels decrease in a statistically significant relationship.

The incongruence between own values and those of the company can erode the worker on a psychological level, not through physical over-exertion, but emotionally and on a daily basis. This phenomenon is common among professions with a strong vocational character (health or education) [31], where there may be a continuous clash between the ideals that initially guided the person towards that activity and the obligation to carry out different activities, particularly those contrary to his or her personal ideals.

An increase in overload rates and a decrease in the congruence of values seem to lead communication professionals in this study towards physical, mental, and emotional burnout, which, according to these results, correlates with the other two dimensions of burnout, DP and PA, and to which the six areas pointed out by Leiter and Maslach also contributed in different ways (depending on the media): workload, control, rewards, community, fairness, and values [17]. These dimensions also showed a statistically significant relationship with health perceptions.

The results point to confirmation of the sequential model of burnout suggested by Leiter, in which workload indices contribute to the EE and this, in turn, to the DP and PA. However, as it has been shown in several studies [10], other working life areas can increase or also moderate these burnout levels, cynicism or lack of personal accomplishment, leading to what has been called “a double process model of burnout”.

Thus, a high workload can be observed in a work environment and yet, high rates of EE may not occur, as would be expected, since other variables such as control over the task, good personal relations, or an adequate adjustment between own values and those of the company, may protect employees. In such a case, and in order to prevent this, these values must be observed and strengthened because if they were to be weakened, the exhaustion process due to overload could be fast and dramatic [9,21].

In the case of professionals in this study, and taking into account the different media in general, it was found that, in addition to workload, as both the index of intrinsic “rewards” of work and the levels in community (a problematic perception in labor relations) decrease, the levels of EE increase. This increase in interpersonal conflict and decrease in rewards also contributed to an increase in the sense of DP, and these same variables, together with low levels of value congruence, generally contribute to a significant decrease in lack of PA. The rewards do not have much to do with the salary received, but rather with the positive feedback for doing a good job, the recognition of bosses, colleagues, and the public. In short, it refers to the feeling that their efforts are worthwhile.

However, these results are not the same for all professionals, nor for all types of media. It was found that overload acted very differently, depending on whether the individual perceived control over their task or not, or whether they felt gratified or comforted by their bosses and colleagues. The overload variable also had different consequences depending on the work environment, or the employees’ perceived sense of community.

The differences found among different types of media (newspapers, radio, TV, institutional press), both in the dimensions of burnout and in the areas that contribute to those values, were statistically significant in the personal accomplishment dimension (PA). Although all of them endured similar workload levels, this did not occur in the areas of control, rewards, community, fairness, and values between the institutional press and the rest of the media (written and audio-visual press), resulting in differences in burnout, especially in personal accomplishment at work.

In subsequent discussions, with professionals from different media to which the results were shown, explanations were given to the fact that journalists, in general, begin their careers with ambitions that are not of an economic nature, but with ideals of telling the truth, contributing to information, change, and perceiving that their work has meaning.

Over the years, many have the feeling of “filling in the pages”, or “telling irrelevant stories”, or they even write or broadcast content contrary to their convictions or vision of reality. On the other hand, those who work for the institutional press have fitted their vocation into a political, social, or economic project. They have accepted this position because there is an affinity in values and goals and, therefore, it is not a problem for them to invest more time or effort in the activities, as they work towards a common goal with their superiors.

Enough scientific support has not been found on this matter regarding communication professionals [32,33], although we consider that it could be extrapolated to other professions. Regardless of salary, sharing the same project with the company, superiors, and colleagues mitigates the harmful effects of burnout very significantly and contributes to personal accomplishment at work [10].

Congruence in values, along with the areas of rewards, justice, and community, become particularly relevant when designing programs for the implementation of a permanent prevention culture. If possible, in addition to analyzing and restructuring the workload in each section and in each position, it is crucial to design the task so that the employee can have maximum control over it. Likewise, it is necessary to assess the existence, or not, of intrinsic rewards, considering the motivational power of providing fair feedback, as well as to review the justice system in the company, tacit or manifest, and to work constantly, not only to promote a good working environment but also for a spirit of success focused on the team and not on individual competition. Finally, the company’s values must be clearly expressed and must show the congruence between those values and what is done daily.

The reality experienced by the media does not facilitate the preventive actions described. These types of companies, in different countries, repeatedly change hands, being owned by different socio-economic groups, and move away from the ideals for which they were created [33]. As we have witnessed in this study, in many of them there is a significant reduction in their workforce, overloading those who remain with responsibility, or there have been shifts in their ideals, leaving no room to choose between keeping the job or defending values.

It is complex to design an intervention program that reduces workload levels and encourages the distribution of rewards or establishes a fair and transparent rules system [34]. It would be even more difficult trying to match a company’s values with those of its employees. For this reason, the preventive program focuses not so much on changing some objectives or goals but on making them clear and manifest so that each person is aware and can choose between supporting them or abandoning them.

However, as previously indicated, this first part of the study was aimed at observing the main psychosocial risks that can emerge in this profession and to develop a model of permanent preventive culture involving employees and managers. The changes introduced in this phase are related to the rationalization of the workload, increased control by the employee over the task, the promotion of team culture, as well as a transparent policy in terms of the values pursued, a system of rewards, and fair mechanisms of appeal. It is expected that the intervention is carried out in a newspaper, a radio station and a television channel will show positive results with regards to its control groups, and that such positive results will be maintained 18 months after its implementation. These kinds of interventions are being promoted by governments and companies because the efforts made are outweighed by the personal, collective, and economic benefits over the medium to long term. Thus, meetings between managers and preventionists are increasingly common to discuss successful experiences that can be applied to different types of working environments [34].

This study has strengths but also limitations. The main strength is the work sector analyzed but the main limitations of this study are the following: it was a cross-sectional study; the data were collected through a self-reported questionnaire; interested individuals/volunteers were recruited in this study, which could represent a possible selection bias, however, this is the most common procedure in this type of study. Furthermore, there was a lack of detailed information about mental health comorbidities such as anxiety and depression, but the aim of the study was not to diagnose but to relate the dimensions of burnout with symptoms of psychopathology (anxiety, depression, social dysfunction) in a population of healthy employees and GHQ-12 is a widely used instrument for mental health disorders screening. Finally, the sample belongs to a group from a specific region in Spain. Therefore, in future studies, the data should be contrasted with those of other countries. Taking into account such limitations, this study is considered to be an approximation to the psychosocial factors experienced by the journalism profession, as well as a necessary analysis to take into consideration when developing a preventive program, whose efficacy shall be shown by the results and contrasted with the data obtained in larger studies.

## 5. Conclusions

Communication professionals suffer, as in other professions, high levels of EE, mainly due to work overload. It can also be claimed that, over time, these employees may be prone to a lack of personal accomplishment at work. However, the most distinctive feature compared with other work environments could be the clash between the aspirations and objectives of the employees and the values genuinely defended by their company daily.

## Figures and Tables

**Table 1:** Description of the characteristics of the sample. Sociodemographic, labor, burnout, and work environment variables.

	Percentages/ Median (IQ Range)	Ranks
Profession		
Executive	10.0%	
Writing, screenwriter	39.5%	
Reporter	14.4%	
Editor/presenter	16.7%	
Photographer, cameraman	4.9%	
Layout designer	0.8%	
Publicist	1.9%	
Administration	2.7%	
Documentalist	3.3%	
Others	5.7%	
Media		
Newspapers	26.4%	
Radio	17.4%	
Television	32.1%	
Institutional Press	24.2%	
Health perception	2 (0)	1–4
GHQ (General Health Questionnaire)	24 (7)	0–36
EE (Emotional Exhaustion)	17 (15)	0–54
DP (Depersonalization)	6 (5)	0–18
PA (Personal Accomplisment)	7 (5)	0–24
Workload	16 (8)	6–30
Control	9 (5)	3–15
Rewards	14 (5)	4–20
Community	17 (5)	5–25
Fairness	17 (9)	6–30
Values	16 (7)	5–25

IQ Range: interquartile range.

**Table 2:** Correlations (Spearman’s Rho) between the dimensions of Emotional Exhaustion (EE), Depersonalization (DP), and Personal Accomplishment (PA)(burnout), Six Areas of Worklife, and personal and health variables.

	EE	DP	PA
Age	−0.068	−0.037	−0.065
Time worked (years)	0.020	0.112	0.075
Health perception	0.215 **	0.174 **	0.130 *
GHQ	0.645 **	0.418 **	0.528 **
DP	0.468 **		0.688 **
PA	0.688 **	0.490 **	
Workload	−0.588 **	−0.080	−0.364 **
Control	−0.516 **	−0.356 **	−0.487 **
Rewards	−0.552 **	−0.458 **	−0.529 **
Community	−0.504 **	−0.399 **	−0.495 **
Fairness	−0.513 **	−0.324 **	−0.512 **
Values	−0.567 **	−0.354 **	−0.576 **

* *p* < 0.05, ** *p* < 0.01; N = 292.

**Table 3:** Linear regression model, taking as dependent variables: EE, DP, and PA and as independent variables the Six Areas of Work Life.

EE	Coefficient	*p*-Value	Confidence Interval 95%	
			Lower	Upper
Constant	59.588	<0.001	54.74	64.42
Workload	−0.945	<0.001	−1.14	−0.74
Rewards	−0.655	<0.001	−0.99	−0.31
Values	−0.556	<0.001	−0.82	−0.28
Community	−0.465	0.002	−0.76	−0.16
R2	0.580			
R2adj	0.573			
**DP**	**Coefficient**	***p*** **-Value**	**Confidence Interval 95%**	
			**Lower**	**Upper**
Constant	13.822	<0.001	12.03	15.60
Rewards	−0.307	<0.001	−0.43	−0.17
Community	−0.193	0.001	−0.31	−0.07
R2	0.240			
R2adj	0.234			
**PA**	**Coefficient**	***p*** **-Value**	**Confidence Interval 95%**	
			**Lower**	**Upper**
Constant	25.853	<0.001	23.15	28.54
Values	−0.399	<0.001	−0.54	−0.25
Rewards	−0.330	0.001	−0.51	−0.14
Community	−0.265	0.002	−0.42	−1.00
Workload	−0.163	0.005	−0.27	−0.05
R2	0.475			
R2adj	0.466			

**Table 4:** Comparison of medians according to the work environment of the variables of perception of health, burnout, and Six Areas of Work Life.

	Press	Radio	TV	Institutional Press	*p*-Value
	Median (IQ)	Median (IQ)	Median (IQ)	Median (IQ)	
Health perception	2 (0)	2 (0)	2 (0)	2 (0)	0.655
GHQ12	24 (8)	25 (8.5)	24 (6)	23 (6.25)	0.151
EE	16 (16)	20 (17.75)	17 (13.75)	13.5 (13)	0.127
DP	6 (5)	7 (5.5)	6.5 (5)	5.5 (6)	0.160
PA	8 (7.25)	9 (6.75)	7 (4)	5 (8)	0.010 *
Workload	16 (8.25)	16 (9)	15 (7.5)	17 (9)	0.506
Control	9 (6)	9.5 (3.75)	9 (5)	11 (4)	0.020 *
Rewards	13 (6)	13 (5)	13 (6)	16 (5)	0.011 *
Comnunity	16.5 (5.25)	18 (6)	16 (4.5)	18 (4.5)	0.016 *
Fairness	16 (7.5)	17.5 (8.5)	15 (8.5)	19 (9)	0.006 **
Values	15 (7)	16 (6)	14 (6)	19 (5)	<0.001 **

IQ: interquartile range. Statistic used: Kruskal-Wallis. * *p* < 0.05, ** *p* < 0.01.

## Data Availability

The data that support the findings of this study are available from the corresponding author, (B.O.-B.), upon reasonable request.
